# Carriage and Genetic Diversity of Methicillin-Resistant *Staphylococcus aureus* among Patients and Healthcare Workers in a Serbian University Hospital

**DOI:** 10.1371/journal.pone.0127347

**Published:** 2015-05-20

**Authors:** Ivana Cirkovic, Srdjan Stepanovic, Robert Skov, Jasmina Trajkovic, Anita Grgurevic, Anders Rhod Larsen

**Affiliations:** 1 Institute of Microbiology and Immunology, Faculty of Medicine, University of Belgrade, Belgrade, Serbia; 2 Department of Microbiological Surveillance and Research, Statens Serum Institut, Copenhagen, Denmark; 3 Institute of Epidemiology, Faculty of Medicine, University of Belgrade, Belgrade, Serbia; Rockefeller University, UNITED STATES

## Abstract

**Objectives:**

There is a paucity of data on methicillin-resistant *Staphylococcus aureus* (MRSA) epidemiology among Balkan countries. The aim of our study was to determine the prevalence of nasal and pharyngeal carriages and diversity of MRSA among patients and healthcare workers (HCWs) in the major referral centre in Serbia, and to evaluate performance of three different media for MRSA screening.

**Methods:**

Nasal and pharyngeal swabs were obtained from 195 patients and 105 HCWs in Emergency Department (ED), Surgical Department (SD) and Medical Department (MD). After broth enrichment, samples were inoculated onto MRSA-ID, ORSA and oxacillin-MSA and incubated for 24/48 hours. Characterisation of isolated MRSA strains was determined by MLVA, *spa*, SCC*mec* and *agr* typing, PVL genes detection and antimicrobial susceptibility.

**Results:**

MRSA carriage prevalence was 11.8% in patients and 7.6% in HCWs. Introduction of pharyngeal swabs in screening procedure increased MRSA carriage rate by over 30%. Variable found to be independently associated with an increased risk for MRSA carriage was ED (odd ratio (OR) = 4.45, 95% confidence interval (CI) 1.78-11.14). A higher risk of multidrug-resistant MRSA carriage was observed among patients (OR = 22; 95% CI 1.92-251.54). CC5-MRSA-SCC*mec*I was the dominant clone among patients and HCWs in ED and MD, while high genetic diversity of community-associated MRSA (CA-MRSA) was shown in SD especially among HCWs. MRSA-ID was superior to the other tested media with a sensitivity/specificity of 95.2% and 99.6% after 48 hours of incubation.

**Conclusions:**

These results indicate high MRSA carriage rate in the hospital and emergence of CA-MRSA through HCWs in these settings. MRSA-ID was the optimal available choice for MRSA screening.

## Introduction

Methicillin-resistant *Staphylococcus aureus* (MRSA) is one of the most significant healthcare-associated (HA) and community-associated (CA) pathogen, causing a wide range of infections, from mild skin and soft tissue infections to life-threatening invasive diseases. The MRSA infections are associated with higher morbidity, mortality, prolonged length of hospital stay and higher healthcare costs as compared to methicillin-susceptible staphylococci [1].

Strategies to control the transmission of MRSA in hospitals require baseline knowledge of the prevalence and characteristics of circulating MRSA strains, which can be obtained by active surveillance. Studies of MRSA carriage in the European hospitals have shown large geographic variations in MRSA prevalence [2] and genetic diversity of strains [3]. In Serbia, the National Reference laboratory for staphylococci was established in 2008, and the data presented here are the first surveillance data on MRSA in Serbia since the dissolution of Yugoslavia in 1991.

Screening and rapid identification of MRSA carriage among patients and healthcare workers (HCWs) are essential steps in controlling MRSA spread in hospitals. Use of selective media involving both chromogenic media and preincubation steps has been proposed for accurate isolation of MRSA [4,5]. Many studies have evaluated the performance of chromogenic media in detecting MRSA [4–10], but several of these are laboratory based studies [4,5], i.e. well-defined collections of staphylococci were used for testing, whereas testing of primary specimens may be of more importance [6,7,10].

The aims of the present study were to: (i) provide data of MRSA carriage among hospitalised patients and HCWs in the largest healthcare facility in Serbia, (ii) determine the genetic lineages of the circulating MRSA and (iii) evaluate the performance of selective media for the detection of MRSA in screening sample after selective enrichment.

## Materials and Methods

### Study design, site and population

A cross-sectional study was conducted between November 2008 and January 2009 at the Clinical Centre of Serbia (CCS), which is a university-affiliated 3000-bed tertiary teaching hospital. CCS serves a population of over 2 million and acts as a major referral health facility in Serbia and some neighbouring countries (Bosnia and Herzegovina, Montenegro).

Screening was conducted in the Emergency Department (ED), Surgical Department (SD) and Medical Department (MD), which are situated 100–200 meters apart in separate buildings. Clinical conditions of patients in the ED ranged from various surgical or medical conditions; in the SD, clinical conditions of patients were primarily surgery related; in the MD, clinical conditions of patients were primarily medically related. Participation was voluntary and all participants signed informed consent form prior to their inclusion in the study. Descriptive information regarding participants' age, sex, diagnosis, department and period of hospitalisation was collected. The study protocol was approved by the Ethics Committee of Faculty of Medicine, University of Belgrade, Belgrade, Serbia [no. 013/2008].

### Sampling

For the present investigation specimens were taken from 195 hospitalised patients (mean age was 58.3+14.7; ages ranged from 19 to 84) and 105 HCWs (mean age was 44.1+10.3; ages ranged from 22 to 61). Participants' response rate was 100% in each ward/department.

Samples for each person included two swabs, one taken from both anterior nares and one from the throat. Demographic characteristics of the study population are shown in Tables 1 and S1.

**Table 1:** Demographic characteristics of patients and healthcare workers (HCWs) at the Clinical Centre of Serbia, Serbia.

Characteristics	Level	Patients (n = 195)	HCWs (n = 105)
Sex [n (%)]	Female	78 (40)	100 (95.2)
	Male	117 (60)	5 (4.8)
Department [n (%)]	ED	66 (33.8)	29 (27.6)
	SD	81 (41.6)	59 (56.2)
	MD	48 (24.6)	17 (16.2)
Age group [n (%)]	< 65	138 (70.8)	105 (100)
	> 65	57 (29.2)	0 (0)
Patient underlying diagnosis [n (%)]	Surgical	132 (67.7)	/
	Non-surgical	63 (32.3)	/
Duration of hospitalization [n (%)]	< 7 days	151 (77.4)	/
	> 7 days	44 (22.6)	/

ED, Emergency Department; SD, Surgical Department; MD, Medical Department.

### Culture methods

After the collection, all samples were processed within 2h. Each swab was inoculated in 3 mL of Mueller-Hinton broth (bioMérieux, France) supplemented with 6.5% NaCl, and incubated 24h at 35°C, in atmospheric air. A 50 μl amount of the culture was inoculated onto chromogenic media MRSA-ID (bioMérieux, France), oxacillin resistance screening agar (ORSA; HiMedia, India), and mannitol salt agar (MSA; bioMérieux, France) supplemented with 2 mg/L of oxacillin, which represent the available MRSA-screening media on the Serbian market. All inoculated plates were incubated at 35°C, in air, and read after 24h and 48h of incubation.

### Identification of isolates

In accordance with the manufacturers’ instructions, a colony suggestive of MRSA was subcultured onto Columbia agar with 5% sheep blood (bioMérieux, France), and later confirmed by PCR for *nuc* [11] and *mecA* [12] genes. One isolate per patient/HCW was further tested.

### Antimicrobial susceptibility testing

Susceptibility to cefoxitin, chloramphenicol, ciprofloxacin, clindamycin, fusidic acid, erythromycin, gentamicin, kanamycin, linezolid, mupirocin, quinupristin-dalfopristin, penicillin, rifampin, tetracycline, tobramycin and trimethoprim/sulfamethoxazole (BioRad, USA) was performed by disk diffusion method and to vancomycin and teicoplanic by Etest (bioMérieux, France) in accordance to the European Committee on Antimicrobial Susceptibility Testing (EUCAST) recommendation (http://www.eucast.org). Multidrug resistance (MDR) was defined as resistance of MRSA to three or more district antimicrobial classes in addition to beta-lactams.

### Molecular characterisation

Multiple-locus variable-number tandem-repeat assay (MLVA) was performed as previously described [13]. Representatives for each MLVA type were subjected to *spa* typing as previously described [14]. The *spa* types were annotated into Multi Locus Sequence Typing (MLST) Clonal complexes (CCs) based on the *spa* repeat successions, information stored in the ridom and MLST database and literature searches (http://www.ridom.de, http://www.mlst.net/).

Determination of Staphylococcal Cassette Chromosome *mec* (SCC*mec*) types and *agr* type were done by previously described multiplex PCR [15,16]. *S*. *aureus* strains HT20020290 (SCC*mec* type I), HT20020285 (SCC*mec* type II), HT20030826 (SCC*mec* type III), HT20040068 (SCC*mec* type IV), HT20060580 (SCC*mec* type V), HT20020274 (SCC*mec* type VI), RN6390 (*agr* type 1), RN6607 (*agr* type 2), RN8465 (*agr* type 3) and RN4850 (*agr* type 4) served as positive controls.

Presence of Panton-Valentine leukocidin (PVL) encoding genes was determined by previously described PCR protocol [17]. *S*. *aureus* ATCC 49775 was used as the positive control.

### Statistical analysis

Descriptive statistics, chi-square test and logistic regression were applied in data analysis using SPSS 21+ statistical software. Univariate logistic regression was used to analyse the association between each risk variable (patient or HCW status, sex, age group, department, patient underlying diagnosis, duration of hospitalisation) and two outcomes: MRSA carriage and MDR MRSA carriage. Based on this model, variables associated with the outcome (MRSA carriage) at a level of significance *P* < 0.1 were entered into the final model of the multivariate logistic regression, which was used to compute adjusted odds ratios (OR) and 95% confidence intervals (95% CI) to assess the independent associations of these variables with the outcome. A forward wald method was selected for this model. Variables remained in multivariate logistic regression analysis if they were independently associated with MRSA carriage at significance level of *P* < 0.05.

## Results

### MRSA carriage

A total of 31/300 (10.3%) participants in the study carried MRSA. Among hospitalised patients and HCWs MRSA carriage rate was 23/195 (11.8%) and 8/105 (7.6%) respectively. Distribution of MRSA carriers and non-carriers stratified by population characteristics are shown in S1 Table and Table 2. Even though patients had higher risk of being carriers than HCWs [OR = 1.62, 95% CI 0.70–3.76] this difference was not significant (*P* = 0.261). Patients in ED [OR = 2.61, 95% CI 1.32–5.17; *P* = 0.006] were found to have higher risk of being MRSA carriers. The variable shown to be independently associated with an increased risk for MRSA carriage was ED hospital department [OR 4.45; 95% CI, 1.78–11.14; *P* = 0.001].

**Table 2:** Distribution of methicillin-resistant *Staphylococcus aureus* (MRSA) carriers and non-carriers among patients and healthcare workers (HCWs) stratified by department, sex, age group, patient underlying diagnosis and duration of hospitalisation at the Clinical Centre of Serbia, Serbia.

Stratifier		Patients and HCWs (n = 300)			Patients (n = 195)			HCWs (n = 105)		
	Group	Carrier	Non-carrier	P*	Carrier	Non-carrier	P*	Carrier	Non-carrier	P*
Sex [n (%)]	Female	17 (9.5)	161 (90.5)	0.591	9 (11.5)	69 (88.5)	0.928	8 (8)	92 (92)	0.511
	Male	14 (11.5)	105 (88.5)		14 (12)	103 (88)		0 (0)	5 (100)	
Department [n (%)]	ED	17 (17.9)	78 (82.1)	0.011	15 (22.7)	51 (77.3)	0.003	2 (6.9)	27 (93.1)	0.374
	SD	11 (7.8)	130 (92.2)		5 (6.2)	76 (93.8)		6 (10.2)	53 (89.8)	
	MD	3 (4.6)	62 (95.4)		3 (6.3)	45 (93.7)		0 (0)	17 (100)	
Age group	< 65	25 (10.3)	218 (89.7)	0.958	17 (12.3)	121 (87.7)	0.724	8 (7.6)	97 (92.4)	/
	> 65	6 (10.5)	51 (89.5)		6 (10.5)	51 (89.5)		/	/	
Patient underlying diagnosis	Surgical	16 (11.5)	116 (88.5)	0.838	16 (11.5)	116 (88.5)	0.838	/	/	/
	Non-surgical	7 (11.1)	56 (88.9)		7 (11.1)	56 (88.9)		/	/	
Duration of hospitalization[n (%)]	< 7 days	21 (13.9)	130 (86.1)	0.090	21 (13.9)	130 (86.1)	0.090	/	/	/
	> 7 days	2 (5.5)	42 (94.5)		2 (5.5)	42 (94.5)		/	/	

* chi-square statistic test; ED, Emergency Department; SD, Surgical Department; MD, Medical Department.

The prevalence of MRSA nasal and throat and just nasal or throat carrier was similar. The presence of MRSA in throat swabs was higher among patients but for nasal swabs the prevalence was similar between patients and HCWs (Table 3).

**Table 3:** Presence of methicillin-resistant *Staphylococcus aureus* (MRSA) in nasal and throat swab specimens obtained from patients and healthcare workers (HCWs) at the Clinical Centre of Serbia, Belgrade.

Result for		Number (%) of MRSA carriers		
Nasal specimen	Throat specimen	Patients	HCWs	Patients and HCWs
Positive	Positive	9 (4.6)	2 (1.9)	11 (3.7)
Positive	Negative	6 (3.1)	4 (3.8)	10 (3.3)
Negative	Positive	8 (4.1)	2 (1.9)	10 (3.3)
Negative	Negative	172 (88.2)	97 (92.4)	269 (89.7)
Total positive		23 (11.8)	8 (7.6)	31 (10.3)

### Antimicrobial susceptibility

Susceptibility testing on one isolate from each MRSA carrier revealed that 29/31 (93.5%) MRSA isolates were resistant to one or more antibiotics in addition to beta-lactam antibiotics, while two isolates (from HCWs) were only resistant to beta-lactams. MDR was noted in 26 (83.9%) isolates. Patients had a higher risk of MDR MRSA carriage compared with HCWs [OR = 22; 95% CI 1.92–251.54; *P* = 0.013]. MDR MRSA carriage was not associated with sex, age group, patient underlying diagnosis, department or duration of hospitalisation.

High levels of resistance among isolated MRSA strains were noted for aminoglycosides (gentamicin: 87.1%, kanamycin 90.3% and tobramycin 87.1%), ciprofloxacin (80.6%) and erythromycin and clindamycin (54.8%) with only two strains exhibiting inducible resistance, whereas resistance to chloramphenicol was 16.1%, tetracycline 12.9%, rifampin 9.7% and fusidic acid 3.2%. All MRSA isolates were susceptible to linezolid, mupirocin, quinupristin-dalfopristin, teicoplanin, trimethoprim/sulfamethoxazole and vancomycin.

Antibiograms were generally well-correlated with the MLVA and *spa* type results except for the MLVA type E clade (*spa* type *t*595, ST152/ST377) where strains exhibited various antibiograms (Table 4).

**Table 4:** Characteristics of 31 methicillin-resistant *Staphylococcus aureus* (MRSA) strains isolated from nasal and throat swab specimens obtained from patients and healthcare workers (HCWs) at the Clinical Centre of Serbia, Serbia.

CC	MLVA type (number of strains patients /HCWs)	*spa* type	*spa* repeats succession	SCC*mec* type	*agr* type	PVL positive strains	Resistance profile (>50% of isolates)
5	A (13/1)	*t*001	26-30-17-34-17-20-17-12-17-16	I	2	0	GEN, KAN, TOB, ERY, CLI, CIP
	B (7/1)	*t*041	26-30-17-34-17-20-17-34-17-20-17-12-17-16	I	2	0	GEN, KAN, TOB, CIP
	C (0/2)	*t*242	26-23-17-13-17-20-17-12-17-16	V	2	0	GEN, KAN, TOB, CHL
8	D (2/1)	*t*030	15-12-16-02-24-24	III	1	0	GEN, KAN, TOB, ERY, CLI, CIP, RIF, TET
ST152/377	E (1/1)	*t*595	07-56-12-17-16-16-33-31-57-31-57-12	V	1	1 (patient)	GEN, KAN, TOB, TET, CHL & other strain was susceptible to all tested antibiotics except beta-lactams
22	F (0/1)	*t*005	26-23-13-23-31-05-17-25-17-25-16-28	V	1	0	susceptible to all tested antibiotics except beta-lactams
80	G (0/1)	*t*044	07-23-12-34-34-33-34	IV	3	1 (HCW)	KAN, FA

CC, clonal complex; MLVA, Multiple-locus variable-number tandem-repeat assay; SCC*mec*, staphylococcal chromosome cassette *mec*; PVL, Panton–Valentine leukocidin; GEN, gentamicin; KAN, kanamycin; TOB, tobramycin; ERY, erythromycin; CLI, clindamycin; CIP, ciprofloxacin; RIF, rifampin; TET, tetracycline; CHL, chloramphenicol; FA, fusidic acid.

### Molecular typing

Based on MLVA typing the MRSA could be divided into seven different MLVA types (A-G), which were annotated to CC group by using *spa* typing. The largest group consisted of isolates belonging to CC5-MRSA-SCC*mec*I including 22/31 (71%) isolates. The CC5-MRSA-SCC*mec*I isolates were predominantly found in patients 20/23 (86.9%), whereas a larger diversity was found among HCW (Table 4). MRSA isolates obtained from HCWs primarily contained the smaller SCC*mec* elements type IV and V, among these the European CA-MRSA clone ST80-MRSA-SCC*mec*IV found in one HCW. This isolate was together with a *t*595 (ST152/ST377) isolate from a patient the only one to contain the PVL gene.

Patients had a higher risk of being colonised with HA-MRSA and HCWs with CA-MRSA [OR = 36.67; 95% CI 3.12–430.33; *P* = 0.004].

Among identified MRSA clones, the CC5-MRSA-SCC*mec*I was predominant in ED (88.2%) and MD (66.7%), while different genetic lineages were detected in SD: CC5-MRSA-SCC*mec*I (45.4%), CC8-MRSA-SCC*mec*III (18.2%), CC5-MRSA-SCC*mec*V (9.1%), CC22-MRSA-SCC*mec*V (9.1%), CC80-MRSA-SCC*mec*IV (9.1%) and CC152-MRSA-SCC*mec*V (9.1%).

### Evaluation of MRSA screening media

Evaluation of selective MRSA media was based on a total of 42/600 swabs, from where MRSA was grown (confirmed by *mecA* and *nuc* PCRs) (S2 Table). MRSA-ID agar was superior to the other tested media and detected MRSA with a sensitivity/specificity of 95.2% and 99.6% after 48 hours of incubation. Prolonged incubation highly improved sensitivity of all media but had a slightly negative impact on the specificity. Table 5 summarises the results of the comparison of the different media used in the experiment.

**Table 5:** Comparison of MRSA-ID, ORSA and oxacillin-MSA in detection of methicillin-resistant *Staphylococcus aureus* (MRSA) verified by *mecA* and *nuc* PCR.

Medium	Number of MRSA strains detected	Sensitivity (%)		Specificity (%)	
	(number after 48h)	24 h	48 h	24 h	48 h
MRSA-ID	34 (40)	80.9	95.2	100	99.6
ORSA	29 (35)	69.1	83.3	99.1	95.1
oxacillin-MSA	27 (33)	64.3	78.6	98.7	94.4
Any*	42 from 600 swabs				

*Any = Total number of MRSA isolated.

It was noticeable that all MRSA isolates belonging to MVLA type C (CC5-MRSA-SCC*mec*V, *spa* type *t*242) and MVLA type E (ST152/ST377-MRSA-SCC*mec*V, *spa* type *t*595) grew only on MRSA-ID media, but did not on ORSA and/or oxacillin-MSA agar.

## Discussion

Emergence, spread and evolution of MRSA are all important aspects of understanding of MRSA epidemiology on a global scale. In the last decade MRSA clones seem to have been spreading more rapidly between continents and countries most likely due to increasing travel activities. To the best of our knowledge, this is the first comprehensive survey of MRSA carriage conducted in Serbia, and thereby provides data from a black spot on the European MRSA map. With the increasing travel activity, detailed knowledge is significant for monitoring and understanding the epidemiology of emerging MRSA clones.

The study of 195 hospitalised patients and 105 HCWs from the largest healthcare facility in our country showed that the prevalence of MRSA in this population was 10.3%. This frequency is high compared to low prevalence countries in Northern Europe and the Netherlands [18] and resemble more what has been reported from high prevalence countries [19,20]. Among Balkan countries, only partial data have been reported from Croatia [21] and Greece [22] with carriage rate ranging from 5.2 to 20%.

The anterior nares are considered to be the primary colonisation site of *S*. *aureus* [23], and therefore most screening programs require obtaining of a swab specimen from the anterior nares only. However, it has been reported that the throat may be selectively colonised by MRSA in significant number, 12.8–20% [24,25]. Screening of the throat in addition to nasal swabs has increased the sensitivity of detection of *S*. *aureus* among carriers by 20% to 26% [2]. In our study among MRSA carriers, 32.2% had MRSA only in nares, 32.2% only in throat and 35.5% in both nares and throat. Therefore, exclusion of throat cultures would have resulted in failure to recognise a substantial part, in our case almost 1/3 of the MRSA carriers.

Results from the current study showed that the prevalence of MRSA carriage was higher among hospitalised patients (11.8%) compared to HCWs (7.6%), and reveal association of department with carriage rate. The results of MRSA carriage may vary significantly between different studies and may be due to true difference in MRSA prevalence in the investigated populations. However, differences in study design, like duration of a study, investigation of particular populations (e.g. patients on dialysis), investigation during epidemics, investigation in a particular units (e.g. ICU), media used for isolation of MRSA, time of incubation of media, may have significant impact on obtained results. MRSA carriage among patients was reported to be 5.9% to 15.6% in nares and 10% to 23.1% in throat [8,9]. In the largest review of studies evaluating the MRSA carriage rate among HCWs, mean nasal carriage was 4.1% in 104 studies, ranging from 0% to 59% and data for MRSA pharyngeal carriage among HCWs were reported only in a few studies, and it was 0.3% [25]. In our study among hospitalized patients and HCWs, nasal carriage rate was 7.7% and 5.7% respectively, and throat carriage rate was 8.7% and 3.8% respectively.

HA-MRSA are typically multiresistant, *agr* types 1 or 2, and may carry *mec*A in many types of SCC*mec* elements of which types I, II or III are found in some of the older clonotypes (eg. CC5-MRSA-SCC*mec*I, ST36-MRSA-SCC*mec*II and ST239-MRSA-SCC*mec*III) whereas SCC*mec*IV is found in eg. ST22-MRSA-SCC*mec*IV and ST45-MRSA-SCC*mec*IV [3]. CA-MRSA strains typically share a type IV or V SCC*mec* and frequently the PVL locus [26]. The finding of CC5-MRSA-SCC*mec*I MRSA isolates in high numbers in our screening of patients in particular but also among HCWs clearly indicates that this clone is circulating in the hospital. In addition, findings of six diverse typically CA-MRSA isolates, five from HCWs, indicate that HCWs may also introduce MRSA isolates into the hospital. It is noticeable that these types were primarily detected in HCWs, which may indicate that they do not easily spread in the hospital environment with a high antibiotic selective pressure. The finding of a highly susceptible MLVA type E isolate from a HCW and another resistant MLVA type E isolate from a hospitalised patient may however emphasise the risk that rapid adaptations can occur (Table 4).

CC5-MRSA-SCC*mec*I MRSA (*spa* types *t*001 and *t*041) has been previously observed in many European hospitals and is a dominant MRSA clone in the neighbouring countries of Croatia, Hungary and Montenegro [3,27], as is the case in our hospital. In the present study a rare association of PVL-negative CC22-MRSA-SCC*mec*V MRSA was found, which had been previously identified in Saxony [3]. Two MRSA strains belonged to CC152-MRSA-SCC*mec*V and one to CC80-MRSA-SCC*mec*IV, previously identified in Serbia and associated with Balkan countries [28].

Laboratory-based screening for MRSA colonisation of patients and HCWs is a key element in enabling control measures and early therapeutic decisions. Several studies have evaluated the performance of chromogenic media in detecting MRSA in terms of their sensitivity, specificity and speed of detection [4–7, 10]. However, only few studies have been based on primary clinical specimens and direct comparison of results is sometimes difficult due to different techniques of processing the specimens (direct inoculation, broth enrichment in non-selective and selective media) [5–7]. Several studies confirm the importance of broth enrichment for accurate detection of MRSA in clinical sample [5,7] and therefore we decided to use this method for isolation of MRSA in the study.

In our hands MRSA-ID was superior to other tested media. However, to achieve excellent sensitivity results 48h of incubation of MRSA-ID was needed. The same has been noted by other authors [5,7,10]. We observed that two strains that were not recovered on MRSA-ID medium were present low in number on ORSA or oxacillin-MSA medium. Similar observations have been made by others [8]. Prolonged incubation increased sensitivity of other tested media too, but also increased the number of false positive results, which has also been shown by others [6,8,10]. High number of false positive results obtained with ORSA, and MSA may be explained by the fact that identification of *S*. *aureus* in these media is based on hydrolysis of mannitol, and many microorganisms including coagulase negative staphylococci hydrolyze mannitol. In addition, mannitol negative MRSA strains have been reported [29]. We observed one strain that during primary-isolation was mannitol negative on ORSA and MSA. Detection of *S*. *aureus* on MRSA-ID is based on the detection of enzyme alpha-glucosidase, and the strain produced typical green colonies on this medium. However, after the strain was subcultured onto MSA it appeared to be mannitol positive, and that was confirmed by ID 32 STAPH (bioMérieux, France) identification system. The strain belonged to MLVA cluster A (CC5, *spa* type *t*001).

One limitation of our study is testing only two chromogenic selective media (MRSA-ID and ORSA) and not others (BBL CHROMagar MRSA II, Oxoid Brilliance MRSA agar, MRSASelect, etc.) that show equal or superior results than the media tested in this study [6,7]. Ideally, more than one batch of each plate should also have been tested which however was not feasible in this study.

## Conclusions

In summary, we demonstrated a high proportion (10.3%) of MRSA among patients and HCWs in the hospital, which places Serbia in middle to high prevalence in the European perspective. We identified a major circulating CC5-MRSA-SCC*mec*I clone in the hospital as well as a variety of other CA-MRSA isolates among HCWs. Increased focus of infection control seems therefore necessary to protect both patients and HCWs in the hospital. Introduction of a screening procedure should include the pharynx, since as much as 1/3 of MRSA carriers were only positive on this site. In order to improve the identification of MRSA, MRSA-ID was the optimal available choice for isolation of MRSA, although the performance of the medium was better after 48h than after 21-24h of incubation.

## Supporting Information

S1 Table Raw data. Distribution of MRSA carriers and non-carriers stratified by population characteristics and characteristics of MRSA strains isolated from MRSA carriers.
(DOCX)

S2 Table Row data. Presence of methicillin-resistant *Staphylococcus aureus* (MRSA) strains among study population (patients and healthcare workers) in nasal and/or throat samples and growth of MRSA strains on culture media.
(DOCX)
