# Supplementary material for: Carriage and Genetic Diversity of Methicillin-Resistant Staphylococcus aureus among Patients and Healthcare Workers in a Serbian University Hospital
Source: PLoS One. 2015 May 20;10(5):e0127347. doi: 10.1371/journal.pone.0127347 (PMC4439055; doi:10.1371/journal.pone.0127347)
Supplement: S1 Table — Distribution of MRSA carriers and non-carriers stratified by population characteristics and characteristics of MRSA strains isolated from MRSA carriers. (DOCX) [file pone.0127347.s001.docx]

Table S1. Raw data. Distribution of MRSA carriers and non-carriers stratified by population characteristics and characteristics of MRSA strains isolated from MRSA carriers

| Patient/HCW | Sex | Department | Age group (years) | Surgical/  Nonsurgical underlying diseases | Hospitalisation (days) | MRSA carriage status | Resistotype | MVLA type | *spa* type | CC | SCC*mec* type | *agr* type | PVL |  |  |  |  |  |  |  |  |  |  |  |  |  |
| --- | --- | --- | --- | --- | --- | --- | --- | --- | --- | --- | --- | --- | --- | --- | --- | --- | --- | --- | --- | --- | --- | --- | --- | --- | --- | --- |
| P 1 | F | ED | < 65 | S | < 7 | - |  |  |  |  |  |  |  |  |  |  |  |  |  |  |  |  |  |  |  |  |
| P 2 | F | ED | < 65 | S | < 7 | - |  |  |  |  |  |  |  |  |  |  |  |  |  |  |  |  |  |  |  |  |
| P 3 | F | ED | < 65 | S | < 7 | - |  |  |  |  |  |  |  |  |  |  |  |  |  |  |  |  |  |  |  |  |
| P 4 | F | ED | < 65 | S | < 7 | - |  |  |  |  |  |  |  |  |  |  |  |  |  |  |  |  |  |  |  |  |
| P 5 | F | ED | < 65 | S | < 7 | - |  |  |  |  |  |  |  |  |  |  |  |  |  |  |  |  |  |  |  |  |
| P 6 | F | ED | < 65 | S | < 7 | - |  |  |  |  |  |  |  |  |  |  |  |  |  |  |  |  |  |  |  |  |
| P 7 | F | ED | < 65 | S | < 7 | - |  |  |  |  |  |  |  |  |  |  |  |  |  |  |  |  |  |  |  |  |
| P 8 | F | ED | < 65 | S | < 7 | - |  |  |  |  |  |  |  |  |  |  |  |  |  |  |  |  |  |  |  |  |
| P 9 | F | ED | < 65 | S | < 7 | - |  |  |  |  |  |  |  |  |  |  |  |  |  |  |  |  |  |  |  |  |
| P 10 | F | ED | < 65 | S | < 7 | - |  |  |  |  |  |  |  |  |  |  |  |  |  |  |  |  |  |  |  |  |
| P 11 | F | ED | < 65 | S | < 7 | - |  |  |  |  |  |  |  |  |  |  |  |  |  |  |  |  |  |  |  |  |
| P 12 | F | ED | > 65 | S | > 7 | - |  |  |  |  |  |  |  |  |  |  |  |  |  |  |  |  |  |  |  |  |
| P 13 | F | ED | > 65 | S | > 7 | - |  |  |  |  |  |  |  |  |  |  |  |  |  |  |  |  |  |  |  |  |
| P 14 | F | ED | > 65 | S | > 7 | - |  |  |  |  |  |  |  |  |  |  |  |  |  |  |  |  |  |  |  |  |
| P 15 | F | ED | > 65 | S | > 7 | - |  |  |  |  |  |  |  |  |  |  |  |  |  |  |  |  |  |  |  |  |
| P 16 | F | ED | > 65 | S | > 7 | - |  |  |  |  |  |  |  |  |  |  |  |  |  |  |  |  |  |  |  |  |
| P 17 | F | ED | < 65 | NS | < 7 | - |  |  |  |  |  |  |  |  |  |  |  |  |  |  |  |  |  |  |  |  |
| P 18 | F | ED | < 65 | NS | < 7 | - |  |  |  |  |  |  |  |  |  |  |  |  |  |  |  |  |  |  |  |  |
| P 19 | F | ED | < 65 | NS | < 7 | - |  |  |  |  |  |  |  |  |  |  |  |  |  |  |  |  |  |  |  |  |
| P 20 | F | ED | < 65 | NS | < 7 | - |  |  |  |  |  |  |  |  |  |  |  |  |  |  |  |  |  |  |  |  |
| P 21 | F | ED | < 65 | NS | < 7 | - |  |  |  |  |  |  |  |  |  |  |  |  |  |  |  |  |  |  |  |  |
| P 22 | F | ED | < 65 | NS | < 7 | - |  |  |  |  |  |  |  |  |  |  |  |  |  |  |  |  |  |  |  |  |
| P 23 | F | ED | > 65 | NS | > 7 | - |  |  |  |  |  |  |  |  |  |  |  |  |  |  |  |  |  |  |  |  |
| P24 | F | SD | < 65 | S | < 7 | - |  |  |  |  |  |  |  |  |  |  |  |  |  |  |  |  |  |  |  |  |
| P25 | F | SD | < 65 | S | < 7 | - |  |  |  |  |  |  |  |  |  |  |  |  |  |  |  |  |  |  |  |  |
| P26 | F | SD | < 65 | S | < 7 | - |  |  |  |  |  |  |  |  |  |  |  |  |  |  |  |  |  |  |  |  |
| P27 | F | SD | < 65 | S | < 7 | - |  |  |  |  |  |  |  |  |  |  |  |  |  |  |  |  |  |  |  |  |
| P28 | F | SD | < 65 | S | < 7 | - |  |  |  |  |  |  |  |  |  |  |  |  |  |  |  |  |  |  |  |  |
| P29 | F | SD | < 65 | S | < 7 | - |  |  |  |  |  |  |  |  |  |  |  |  |  |  |  |  |  |  |  |  |
| P30 | F | SD | < 65 | S | < 7 | - |  |  |  |  |  |  |  |  |  |  |  |  |  |  |  |  |  |  |  |  |
| P31 | F | SD | < 65 | S | < 7 | - |  |  |  |  |  |  |  |  |  |  |  |  |  |  |  |  |  |  |  |  |
| P32 | F | SD | < 65 | S | < 7 | - |  |  |  |  |  |  |  |  |  |  |  |  |  |  |  |  |  |  |  |  |
| P33 | F | SD | < 65 | S | < 7 | - |  |  |  |  |  |  |  |  |  |  |  |  |  |  |  |  |  |  |  |  |
| P34 | F | SD | < 65 | S | < 7 | - |  |  |  |  |  |  |  |  |  |  |  |  |  |  |  |  |  |  |  |  |
| P35 | F | SD | < 65 | S | < 7 | - |  |  |  |  |  |  |  |  |  |  |  |  |  |  |  |  |  |  |  |  |
| P36 | F | SD | < 65 | S | < 7 | - |  |  |  |  |  |  |  |  |  |  |  |  |  |  |  |  |  |  |  |  |
| P37 | F | SD | < 65 | S | < 7 | - |  |  |  |  |  |  |  |  |  |  |  |  |  |  |  |  |  |  |  |  |
| P38 | F | SD | > 65 | S | > 7 | - |  |  |  |  |  |  |  |  |  |  |  |  |  |  |  |  |  |  |  |  |
| P39 | F | SD | > 65 | S | > 7 | - |  |  |  |  |  |  |  |  |  |  |  |  |  |  |  |  |  |  |  |  |
| P40 | F | SD | > 65 | S | > 7 | - |  |  |  |  |  |  |  |  |  |  |  |  |  |  |  |  |  |  |  |  |
| P41 | F | SD | > 65 | S | > 7 | - |  |  |  |  |  |  |  |  |  |  |  |  |  |  |  |  |  |  |  |  |
| P42 | F | SD | > 65 | S | > 7 | - |  |  |  |  |  |  |  |  |  |  |  |  |  |  |  |  |  |  |  |  |
| P43 | F | SD | > 65 | S | > 7 | - |  |  |  |  |  |  |  |  |  |  |  |  |  |  |  |  |  |  |  |  |
| P44 | F | SD | > 65 | S | > 7 | - |  |  |  |  |  |  |  |  |  |  |  |  |  |  |  |  |  |  |  |  |
| P45 | F | SD | < 65 | NS | < 7 | - |  |  |  |  |  |  |  |  |  |  |  |  |  |  |  |  |  |  |  |  |
| P46 | F | SD | < 65 | NS | < 7 | - |  |  |  |  |  |  |  |  |  |  |  |  |  |  |  |  |  |  |  |  |
| P47 | F | SD | < 65 | NS | < 7 | - |  |  |  |  |  |  |  |  |  |  |  |  |  |  |  |  |  |  |  |  |
| P48 | F | SD | < 65 | NS | < 7 | - |  |  |  |  |  |  |  |  |  |  |  |  |  |  |  |  |  |  |  |  |
| P49 | F | SD | < 65 | NS | < 7 | - |  |  |  |  |  |  |  |  |  |  |  |  |  |  |  |  |  |  |  |  |
| P50 | F | SD | < 65 | NS | < 7 | - |  |  |  |  |  |  |  |  |  |  |  |  |  |  |  |  |  |  |  |  |
| P51 | F | SD | < 65 | NS | < 7 | - |  |  |  |  |  |  |  |  |  |  |  |  |  |  |  |  |  |  |  |  |
| P52 | F | SD | < 65 | NS | < 7 | - |  |  |  |  |  |  |  |  |  |  |  |  |  |  |  |  |  |  |  |  |
| P53 | F | SD | < 65 | NS | < 7 | - |  |  |  |  |  |  |  |  |  |  |  |  |  |  |  |  |  |  |  |  |
| P54 | F | SD | > 65 | NS | > 7 | - |  |  |  |  |  |  |  |  |  |  |  |  |  |  |  |  |  |  |  |  |
| P55 | F | MD | > 65 | S | < 7 | - |  |  |  |  |  |  |  |  |  |  |  |  |  |  |  |  |  |  |  |  |
| P56 | F | MD | > 65 | S | < 7 | - |  |  |  |  |  |  |  |  |  |  |  |  |  |  |  |  |  |  |  |  |
| P57 | F | MD | > 65 | S | < 7 | - |  |  |  |  |  |  |  |  |  |  |  |  |  |  |  |  |  |  |  |  |
| P58 | F | MD | > 65 | S | < 7 | - |  |  |  |  |  |  |  |  |  |  |  |  |  |  |  |  |  |  |  |  |
| P59 | F | MD | > 65 | S | < 7 | - |  |  |  |  |  |  |  |  |  |  |  |  |  |  |  |  |  |  |  |  |
| P60 | F | MD | > 65 | S | < 7 | - |  |  |  |  |  |  |  |  |  |  |  |  |  |  |  |  |  |  |  |  |
| P61 | F | MD | < 65 | S | > 7 | - |  |  |  |  |  |  |  |  |  |  |  |  |  |  |  |  |  |  |  |  |
| P62 | F | MD | < 65 | S | > 7 | - |  |  |  |  |  |  |  |  |  |  |  |  |  |  |  |  |  |  |  |  |
| P63 | F | MD | < 65 | S | > 7 | - |  |  |  |  |  |  |  |  |  |  |  |  |  |  |  |  |  |  |  |  |
| P64 | F | MD | > 65 | NS | < 7 | - |  |  |  |  |  |  |  |  |  |  |  |  |  |  |  |  |  |  |  |  |
| P65 | F | MD | > 65 | NS | < 7 | - |  |  |  |  |  |  |  |  |  |  |  |  |  |  |  |  |  |  |  |  |
| P65 | F | MD | > 65 | NS | < 7 | - |  |  |  |  |  |  |  |  |  |  |  |  |  |  |  |  |  |  |  |  |
| P66 | F | MD | > 65 | NS | < 7 | - |  |  |  |  |  |  |  |  |  |  |  |  |  |  |  |  |  |  |  |  |
| P67 | F | MD | > 65 | NS | < 7 | - |  |  |  |  |  |  |  |  |  |  |  |  |  |  |  |  |  |  |  |  |
| P68 | F | MD | > 65 | NS | < 7 | - |  |  |  |  |  |  |  |  |  |  |  |  |  |  |  |  |  |  |  |  |
| P69 | F | MD | < 65 | NS | > 7 | - |  |  |  |  |  |  |  |  |  |  |  |  |  |  |  |  |  |  |  |  |
| P70 | M | ED | < 65 | S | < 7 | - |  |  |  |  |  |  |  |  |  |  |  |  |  |  |  |  |  |  |  |  |
| P71 | M | ED | < 65 | S | < 7 | - |  |  |  |  |  |  |  |  |  |  |  |  |  |  |  |  |  |  |  |  |
| P72 | M | ED | < 65 | S | < 7 | - |  |  |  |  |  |  |  |  |  |  |  |  |  |  |  |  |  |  |  |  |
| P73 | M | ED | < 65 | S | < 7 | - |  |  |  |  |  |  |  |  |  |  |  |  |  |  |  |  |  |  |  |  |
| P74 | M | ED | < 65 | S | < 7 | - |  |  |  |  |  |  |  |  |  |  |  |  |  |  |  |  |  |  |  |  |
| P75 | M | ED | < 65 | S | < 7 | - |  |  |  |  |  |  |  |  |  |  |  |  |  |  |  |  |  |  |  |  |
| P76 | M | ED | < 65 | S | < 7 | - |  |  |  |  |  |  |  |  |  |  |  |  |  |  |  |  |  |  |  |  |
| P77 | M | ED | < 65 | S | < 7 | - |  |  |  |  |  |  |  |  |  |  |  |  |  |  |  |  |  |  |  |  |
| P78 | M | ED | < 65 | S | < 7 | - |  |  |  |  |  |  |  |  |  |  |  |  |  |  |  |  |  |  |  |  |
| P79 | M | ED | < 65 | S | < 7 | - |  |  |  |  |  |  |  |  |  |  |  |  |  |  |  |  |  |  |  |  |
| P80 | M | ED | < 65 | S | < 7 | - |  |  |  |  |  |  |  |  |  |  |  |  |  |  |  |  |  |  |  |  |
| P81 | M | ED | < 65 | S | < 7 | - |  |  |  |  |  |  |  |  |  |  |  |  |  |  |  |  |  |  |  |  |
| P82 | M | ED | < 65 | S | < 7 | - |  |  |  |  |  |  |  |  |  |  |  |  |  |  |  |  |  |  |  |  |
| P83 | M | ED | < 65 | S | < 7 | - |  |  |  |  |  |  |  |  |  |  |  |  |  |  |  |  |  |  |  |  |
| P84 | M | ED | > 65 | S | > 7 | - |  |  |  |  |  |  |  |  |  |  |  |  |  |  |  |  |  |  |  |  |
| P85 | M | ED | > 65 | S | > 7 | - |  |  |  |  |  |  |  |  |  |  |  |  |  |  |  |  |  |  |  |  |
| P86 | M | ED | > 65 | S | > 7 | - |  |  |  |  |  |  |  |  |  |  |  |  |  |  |  |  |  |  |  |  |
| P87 | M | ED | > 65 | S | > 7 | - |  |  |  |  |  |  |  |  |  |  |  |  |  |  |  |  |  |  |  |  |
| P88 | M | ED | < 65 | NS | < 7 | - |  |  |  |  |  |  |  |  |  |  |  |  |  |  |  |  |  |  |  |  |
| P89 | M | ED | < 65 | NS | < 7 | - |  |  |  |  |  |  |  |  |  |  |  |  |  |  |  |  |  |  |  |  |
| P90 | M | ED | < 65 | NS | < 7 | - |  |  |  |  |  |  |  |  |  |  |  |  |  |  |  |  |  |  |  |  |
| P91 | M | ED | < 65 | NS | < 7 | - |  |  |  |  |  |  |  |  |  |  |  |  |  |  |  |  |  |  |  |  |
| P92 | M | ED | < 65 | NS | < 7 | - |  |  |  |  |  |  |  |  |  |  |  |  |  |  |  |  |  |  |  |  |
| P93 | M | ED | < 65 | NS | < 7 | - |  |  |  |  |  |  |  |  |  |  |  |  |  |  |  |  |  |  |  |  |
| P94 | M | ED | < 65 | NS | < 7 | - |  |  |  |  |  |  |  |  |  |  |  |  |  |  |  |  |  |  |  |  |
| P95 | M | ED | < 65 | NS | < 7 | - |  |  |  |  |  |  |  |  |  |  |  |  |  |  |  |  |  |  |  |  |
| P96 | M | ED | > 65 | NS | > 7 | - |  |  |  |  |  |  |  |  |  |  |  |  |  |  |  |  |  |  |  |  |
| P97 | M | ED | > 65 | NS | > 7 | - |  |  |  |  |  |  |  |  |  |  |  |  |  |  |  |  |  |  |  |  |
| P98 | M | SD | < 65 | S | < 7 | - |  |  |  |  |  |  |  |  |  |  |  |  |  |  |  |  |  |  |  |  |
| P99 | M | SD | < 65 | S | < 7 | - |  |  |  |  |  |  |  |  |  |  |  |  |  |  |  |  |  |  |  |  |
| P100 | M | SD | < 65 | S | < 7 | - |  |  |  |  |  |  |  |  |  |  |  |  |  |  |  |  |  |  |  |  |
| P101 | M | SD | < 65 | S | < 7 | - |  |  |  |  |  |  |  |  |  |  |  |  |  |  |  |  |  |  |  |  |
| P102 | M | SD | < 65 | S | < 7 | - |  |  |  |  |  |  |  |  |  |  |  |  |  |  |  |  |  |  |  |  |
| P103 | M | SD | < 65 | S | < 7 | - |  |  |  |  |  |  |  |  |  |  |  |  |  |  |  |  |  |  |  |  |
| P104 | M | SD | < 65 | S | < 7 | - |  |  |  |  |  |  |  |  |  |  |  |  |  |  |  |  |  |  |  |  |
| P105 | M | SD | < 65 | S | < 7 | - |  |  |  |  |  |  |  |  |  |  |  |  |  |  |  |  |  |  |  |  |
| P106 | M | SD | < 65 | S | < 7 | - |  |  |  |  |  |  |  |  |  |  |  |  |  |  |  |  |  |  |  |  |
| P107 | M | SD | < 65 | S | < 7 | - |  |  |  |  |  |  |  |  |  |  |  |  |  |  |  |  |  |  |  |  |
| P108 | M | SD | < 65 | S | < 7 | - |  |  |  |  |  |  |  |  |  |  |  |  |  |  |  |  |  |  |  |  |
| P109 | M | SD | < 65 | S | < 7 | - |  |  |  |  |  |  |  |  |  |  |  |  |  |  |  |  |  |  |  |  |
| P110 | M | SD | < 65 | S | < 7 | - |  |  |  |  |  |  |  |  |  |  |  |  |  |  |  |  |  |  |  |  |
| P111 | M | SD | < 65 | S | < 7 | - |  |  |  |  |  |  |  |  |  |  |  |  |  |  |  |  |  |  |  |  |
| P112 | M | SD | < 65 | S | < 7 | - |  |  |  |  |  |  |  |  |  |  |  |  |  |  |  |  |  |  |  |  |
| P113 | M | SD | < 65 | S | < 7 | - |  |  |  |  |  |  |  |  |  |  |  |  |  |  |  |  |  |  |  |  |
| P114 | M | SD | < 65 | S | < 7 | - |  |  |  |  |  |  |  |  |  |  |  |  |  |  |  |  |  |  |  |  |
| P115 | M | SD | < 65 | S | < 7 | - |  |  |  |  |  |  |  |  |  |  |  |  |  |  |  |  |  |  |  |  |
| P116 | M | SD | < 65 | S | < 7 | - |  |  |  |  |  |  |  |  |  |  |  |  |  |  |  |  |  |  |  |  |
| P117 | M | SD | < 65 | S | < 7 | - |  |  |  |  |  |  |  |  |  |  |  |  |  |  |  |  |  |  |  |  |
| P118 | M | SD | < 65 | S | < 7 | - |  |  |  |  |  |  |  |  |  |  |  |  |  |  |  |  |  |  |  |  |
| P119 | M | SD | < 65 | S | < 7 | - |  |  |  |  |  |  |  |  |  |  |  |  |  |  |  |  |  |  |  |  |
| P120 | M | SD | < 65 | S | < 7 | - |  |  |  |  |  |  |  |  |  |  |  |  |  |  |  |  |  |  |  |  |
| P121 | M | SD | < 65 | S | < 7 | - |  |  |  |  |  |  |  |  |  |  |  |  |  |  |  |  |  |  |  |  |
| P122 | M | SD | < 65 | S | < 7 | - |  |  |  |  |  |  |  |  |  |  |  |  |  |  |  |  |  |  |  |  |
| P123 | M | SD | > 65 | S | > 7 | - |  |  |  |  |  |  |  |  |  |  |  |  |  |  |  |  |  |  |  |  |
| P124 | M | SD | > 65 | S | > 7 | - |  |  |  |  |  |  |  |  |  |  |  |  |  |  |  |  |  |  |  |  |
| P125 | M | SD | > 65 | S | > 7 | - |  |  |  |  |  |  |  |  |  |  |  |  |  |  |  |  |  |  |  |  |
| P126 | M | SD | > 65 | S | > 7 |  |  |  |  |  |  |  |  |  |  |  |  |  |  |  |  |  |  |  |  |  |
| P127 | M | SD | > 65 | S | > 7 | - |  |  |  |  |  |  |  |  |  |  |  |  |  |  |  |  |  |  |  |  |
| P128 | M | SD | > 65 | S | > 7 | - |  |  |  |  |  |  |  |  |  |  |  |  |  |  |  |  |  |  |  |  |
| P129 | M | SD | > 65 | S | > 7 | - |  |  |  |  |  |  |  |  |  |  |  |  |  |  |  |  |  |  |  |  |
| P130 | M | SD | < 65 | NS | < 7 | - |  |  |  |  |  |  |  |  |  |  |  |  |  |  |  |  |  |  |  |  |
| P131 | M | SD | < 65 | NS | < 7 | - |  |  |  |  |  |  |  |  |  |  |  |  |  |  |  |  |  |  |  |  |
| P132 | M | SD | < 65 | NS | < 7 | - |  |  |  |  |  |  |  |  |  |  |  |  |  |  |  |  |  |  |  |  |
| P133 | M | SD | < 65 | NS | < 7 |  |  |  |  |  |  |  |  |  |  |  |  |  |  |  |  |  |  |  |  |  |
| P134 | M | SD | < 65 | NS | < 7 | - |  |  |  |  |  |  |  |  |  |  |  |  |  |  |  |  |  |  |  |  |
| P135 | M | SD | < 65 | NS | < 7 | - |  |  |  |  |  |  |  |  |  |  |  |  |  |  |  |  |  |  |  |  |
| P136 | M | SD | < 65 | NS | < 7 | - |  |  |  |  |  |  |  |  |  |  |  |  |  |  |  |  |  |  |  |  |
| P137 | M | SD | < 65 | NS | < 7 | - |  |  |  |  |  |  |  |  |  |  |  |  |  |  |  |  |  |  |  |  |
| P138 | M | SD | < 65 | NS | < 7 | - |  |  |  |  |  |  |  |  |  |  |  |  |  |  |  |  |  |  |  |  |
| P139 | M | SD | < 65 | NS | < 7 | - |  |  |  |  |  |  |  |  |  |  |  |  |  |  |  |  |  |  |  |  |
| P140 | M | SD | > 65 | NS | > 7 | - |  |  |  |  |  |  |  |  |  |  |  |  |  |  |  |  |  |  |  |  |
| P141 | M | SD | > 65 | NS | > 7 | - |  |  |  |  |  |  |  |  |  |  |  |  |  |  |  |  |  |  |  |  |
| P142 | M | SD | > 65 | NS | > 7 | - |  |  |  |  |  |  |  |  |  |  |  |  |  |  |  |  |  |  |  |  |
| P143 | M | MD | < 65 | S | < 7 | - |  |  |  |  |  |  |  |  |  |  |  |  |  |  |  |  |  |  |  |  |
| P144 | M | MD | < 65 | S | < 7 | - |  |  |  |  |  |  |  |  |  |  |  |  |  |  |  |  |  |  |  |  |
| P145 | M | MD | < 65 | S | < 7 | - |  |  |  |  |  |  |  |  |  |  |  |  |  |  |  |  |  |  |  |  |
| P146 | M | MD | < 65 | S | < 7 | - |  |  |  |  |  |  |  |  |  |  |  |  |  |  |  |  |  |  |  |  |
| P147 | M | MD | < 65 | S | < 7 | - |  |  |  |  |  |  |  |  |  |  |  |  |  |  |  |  |  |  |  |  |
| P148 | M | MD | < 65 | S | < 7 | - |  |  |  |  |  |  |  |  |  |  |  |  |  |  |  |  |  |  |  |  |
| P149 | M | MD | < 65 | S | < 7 | - |  |  |  |  |  |  |  |  |  |  |  |  |  |  |  |  |  |  |  |  |
| P150 | M | MD | < 65 | S | < 7 | - |  |  |  |  |  |  |  |  |  |  |  |  |  |  |  |  |  |  |  |  |
| P151 | M | MD | < 65 | S | < 7 | - |  |  |  |  |  |  |  |  |  |  |  |  |  |  |  |  |  |  |  |  |
| P152 | M | MD | < 65 | S | < 7 | - |  |  |  |  |  |  |  |  |  |  |  |  |  |  |  |  |  |  |  |  |
| P153 | M | MD | < 65 | S | < 7 | - |  |  |  |  |  |  |  |  |  |  |  |  |  |  |  |  |  |  |  |  |
| P154 | M | MD | < 65 | S | < 7 | - |  |  |  |  |  |  |  |  |  |  |  |  |  |  |  |  |  |  |  |  |
| P155 | M | MD | < 65 | S | < 7 | - |  |  |  |  |  |  |  |  |  |  |  |  |  |  |  |  |  |  |  |  |
| P156 | M | MD | < 65 | S | < 7 | - |  |  |  |  |  |  |  |  |  |  |  |  |  |  |  |  |  |  |  |  |
| P157 | M | MD | < 65 | S | < 7 | - |  |  |  |  |  |  |  |  |  |  |  |  |  |  |  |  |  |  |  |  |
| P158 | M | MD | < 65 | S | < 7 | - |  |  |  |  |  |  |  |  |  |  |  |  |  |  |  |  |  |  |  |  |
| P159 | M | MD | > 65 | S | > 7 | - |  |  |  |  |  |  |  |  |  |  |  |  |  |  |  |  |  |  |  |  |
| P160 | M | MD | > 65 | S | > 7 | - |  |  |  |  |  |  |  |  |  |  |  |  |  |  |  |  |  |  |  |  |
| P161 | M | MD | > 65 | S | > 7 | - |  |  |  |  |  |  |  |  |  |  |  |  |  |  |  |  |  |  |  |  |
| P162 | M | MD | > 65 | S | > 7 | - |  |  |  |  |  |  |  |  |  |  |  |  |  |  |  |  |  |  |  |  |
| P163 | M | MD | > 65 | NS | < 7 | - |  |  |  |  |  |  |  |  |  |  |  |  |  |  |  |  |  |  |  |  |
| P164 | M | MD | > 65 | NS | < 7 | - |  |  |  |  |  |  |  |  |  |  |  |  |  |  |  |  |  |  |  |  |
| P165 | M | MD | > 65 | NS | < 7 | - |  |  |  |  |  |  |  |  |  |  |  |  |  |  |  |  |  |  |  |  |
| P166 | M | MD | > 65 | NS | < 7 | - |  |  |  |  |  |  |  |  |  |  |  |  |  |  |  |  |  |  |  |  |
| P167 | M | MD | > 65 | NS | < 7 | - |  |  |  |  |  |  |  |  |  |  |  |  |  |  |  |  |  |  |  |  |
| P168 | M | MD | > 65 | NS | < 7 | - |  |  |  |  |  |  |  |  |  |  |  |  |  |  |  |  |  |  |  |  |
| P169 | M | MD | < 65 | NS | > 7 | - |  |  |  |  |  |  |  |  |  |  |  |  |  |  |  |  |  |  |  |  |
| P170 | M | MD | < 65 | NS | > 7 | - |  |  |  |  |  |  |  |  |  |  |  |  |  |  |  |  |  |  |  |  |
| P171 | M | MD | < 65 | NS | > 7 | - |  |  |  |  |  |  |  |  |  |  |  |  |  |  |  |  |  |  |  |  |
| P172 | M | MD | < 65 | NS | > 7 | - |  |  |  |  |  |  |  |  |  |  |  |  |  |  |  |  |  |  |  |  |
| P173 | F | ED | < 65 | S | < 7 | + | GEN, KAN, TOB, ERY, CLI, CIP | A | t001 | 5 | I | II | - |  |  |  |  |  |  |  |  |  |  |  |  |  |
| P174 | F | ED | < 65 | S | < 7 | + | GEN, KAN, TOB, ERY, CLI, CIP | A | t001 | 5 | I | II | - |  |  |  |  |  |  |  |  |  |  |  |  |  |
| P175 | F | ED | < 65 | S | < 7 | + | GEN, KAN, TOB, ERY, CLI, CIP | A | t001 | 5 | I | II | - |  |  |  |  |  |  |  |  |  |  |  |  |  |
| P176 | F | ED | > 65 | S | < 7 | + | GEN, KAN, TOB, ERY, CLI, CIP | A | t001 | 5 | I | II | - |  |  |  |  |  |  |  |  |  |  |  |  |  |
| P177 | F | SD | > 65 | S | < 7 | + | GEN, KAN, TOB, ERY, CLI, CIP | A | t001 | 5 | I | II | - |  |  |  |  |  |  |  |  |  |  |  |  |  |
| P178 | F | SD | > 65 | S | < 7 | + | GEN, KAN, TOB, ERY, CLI, CIP | A | t001 | 5 | I | II | - |  |  |  |  |  |  |  |  |  |  |  |  |  |
| P179 | F | MD | > 65 | NS | < 7 | + | GEN, KAN, TOB, CIP | B | t041 | 5 | I | II | - |  |  |  |  |  |  |  |  |  |  |  |  |  |
| P180 | F | MD | > 65 | NS | > 7 | + | GEN, KAN, TOB, CIP | B | t041 | 5 | I | II | - |  |  |  |  |  |  |  |  |  |  |  |  |  |
| P181 | F | SD | < 65 | NS | < 7 | + | GEN, KAN, TOB, CIP | B | t041 | 5 | I | II | - |  |  |  |  |  |  |  |  |  |  |  |  |  |
| P182 | M | ED | < 65 | S | < 7 | + | GEN, KAN, TOB, CIP | B | t041 | 5 | I | II | - |  |  |  |  |  |  |  |  |  |  |  |  |  |
| P183 | M | ED | < 65 | S | < 7 | + | GEN, KAN, TOB, CIP | B | t041 | 5 | I | II | - |  |  |  |  |  |  |  |  |  |  |  |  |  |
| P184 | M | ED | < 65 | S | < 7 | + | GEN, KAN, TOB, ERY, CLI, CIP | A | t001 | 5 | I | II | - |  |  |  |  |  |  |  |  |  |  |  |  |  |
| P185 | M | ED | < 65 | S | < 7 | + | GEN, KAN, TOB, ERY, CLI, CIP | A | t001 | 5 | I | II | - |  |  |  |  |  |  |  |  |  |  |  |  |  |
| P186 | M | ED | < 65 | S | < 7 | + | GEN, KAN, TOB, ERY, CLI, CIP | A | t001 | 5 | I | II | - |  |  |  |  |  |  |  |  |  |  |  |  |  |
| P187 | M | ED | < 65 | S | < 7 | + | GEN, KAN, TOB, ERY, CLI, CIP | A | t001 | 5 | I | II | - |  |  |  |  |  |  |  |  |  |  |  |  |  |
| P188 | M | ED | < 65 | S | < 7 | + | GEN, KAN, TOB, ERY, CLI, CIP | A | t001 | 5 | I | II | - |  |  |  |  |  |  |  |  |  |  |  |  |  |
| P189 | M | ED | < 65 | S | < 7 | + | GEN, KAN, TOB, ERY, CLI, CIP | A | t001 | 5 | I | II | - |  |  |  |  |  |  |  |  |  |  |  |  |  |
| P190 | M | ED | < 65 | NS | < 7 | + | GEN, KAN, TOB, CIP | B | t041 | 5 | I | II | - |  |  |  |  |  |  |  |  |  |  |  |  |  |
| P191 | M | ED | < 65 | NS | < 7 | + | GEN, KAN, TOB, CIP | B | t041 | 5 | I | II | - |  |  |  |  |  |  |  |  |  |  |  |  |  |
| P192 | M | ED | < 65 | NS | < 7 | + | GEN, KAN, TOB, ERY, CLI, CIP, RIF, TET | D | t030 | 8 | III | I | - |  |  |  |  |  |  |  |  |  |  |  |  |  |
| P193 | M | SD | < 65 | S | < 7 | + | GEN, KAN, TOB, TET, CHL | E | t595 | 152 | V | I | + |  |  |  |  |  |  |  |  |  |  |  |  |  |
| P194 | M | SD | < 65 | S | < 7 | + | GEN, KAN, TOB, ERY, CLI, CIP, RIF, TET | D | t030 | 8 | III | II | - |  |  |  |  |  |  |  |  |  |  |  |  |  |
| P195 | M | MD | > 65 | NS | > 7 | + | GEN, KAN, TOB, ERY, CLI, CIP | A | t001 | 5 | I | I | - |  |  |  |  |  |  |  |  |  |  |  |  |  |
| HCW1 | M | ED | < 65 | / | / | - |  |  |  |  |  |  |  |  |  |  |  |  |  |  |  |  |  |  |  |  |
| HCW2 | M | ED | < 65 | / | / | - |  |  |  |  |  |  |  |  |  |  |  |  |  |  |  |  |  |  |  |  |
| HCW3 | M | ED | < 65 | / | / | - |  |  |  |  |  |  |  |  |  |  |  |  |  |  |  |  |  |  |  |  |
| HCW4 | M | ED | < 65 | / | / | - |  |  |  |  |  |  |  |  |  |  |  |  |  |  |  |  |  |  |  |  |
| HCW5 | M | ED | < 65 | / | / | - |  |  |  |  |  |  |  |  |  |  |  |  |  |  |  |  |  |  |  |  |
| HCW6 | F | ED | < 65 | / | / | - |  |  |  |  |  |  |  |  |  |  |  |  |  |  |  |  |  |  |  |  |
| HCW7 | F | ED | < 65 | / | / | - |  |  |  |  |  |  |  |  |  |  |  |  |  |  |  |  |  |  |  |  |
| HCW8 | F | ED | < 65 | / | / | - |  |  |  |  |  |  |  |  |  |  |  |  |  |  |  |  |  |  |  |  |
| HCW9 | F | ED | < 65 | / | / | - |  |  |  |  |  |  |  |  |  |  |  |  |  |  |  |  |  |  |  |  |
| HCW10 | F | ED | < 65 | / | / | - |  |  |  |  |  |  |  |  |  |  |  |  |  |  |  |  |  |  |  |  |
| HCW11 | F | ED | < 65 | / | / | - |  |  |  |  |  |  |  |  |  |  |  |  |  |  |  |  |  |  |  |  |
| HCW12 | F | ED | < 65 | / | / | - |  |  |  |  |  |  |  |  |  |  |  |  |  |  |  |  |  |  |  |  |
| HCW13 | F | ED | < 65 | / | / | - |  |  |  |  |  |  |  |  |  |  |  |  |  |  |  |  |  |  |  |  |
| HCW14 | F | ED | < 65 | / | / | - |  |  |  |  |  |  |  |  |  |  |  |  |  |  |  |  |  |  |  |  |
| HCW15 | F | ED | < 65 | / | / | - |  |  |  |  |  |  |  |  |  |  |  |  |  |  |  |  |  |  |  |  |
| HCW16 | F | ED | < 65 | / | / | - |  |  |  |  |  |  |  |  |  |  |  |  |  |  |  |  |  |  |  |  |
| HCW17 | F | ED | < 65 | / | / | - |  |  |  |  |  |  |  |  |  |  |  |  |  |  |  |  |  |  |  |  |
| HCW18 | F | ED | < 65 | / | / | - |  |  |  |  |  |  |  |  |  |  |  |  |  |  |  |  |  |  |  |  |
| HCW19 | F | ED | < 65 | / | / | - |  |  |  |  |  |  |  |  |  |  |  |  |  |  |  |  |  |  |  |  |
| HCW20 | F | ED | < 65 | / | / | - |  |  |  |  |  |  |  |  |  |  |  |  |  |  |  |  |  |  |  |  |
| HCW21 | F | ED | < 65 | / | / | - |  |  |  |  |  |  |  |  |  |  |  |  |  |  |  |  |  |  |  |  |
| HCW22 | F | ED | < 65 | / | / | - |  |  |  |  |  |  |  |  |  |  |  |  |  |  |  |  |  |  |  |  |
| HCW23 | F | ED | < 65 | / | / | - |  |  |  |  |  |  |  |  |  |  |  |  |  |  |  |  |  |  |  |  |
| HCW24 | F | ED | < 65 | / | / | - |  |  |  |  |  |  |  |  |  |  |  |  |  |  |  |  |  |  |  |  |
| HCW25 | F | ED | < 65 | / | / | - |  |  |  |  |  |  |  |  |  |  |  |  |  |  |  |  |  |  |  |  |
| HCW26 | F | ED | < 65 | / | / | - |  |  |  |  |  |  |  |  |  |  |  |  |  |  |  |  |  |  |  |  |
| HCW27 | F | ED | < 65 | / | / | - |  |  |  |  |  |  |  |  |  |  |  |  |  |  |  |  |  |  |  |  |
| HCW28 | F | SD | < 65 | / | / | - |  |  |  |  |  |  |  |  |  |  |  |  |  |  |  |  |  |  |  |  |
| HCW29 | F | SD | < 65 | / | / | - |  |  |  |  |  |  |  |  |  |  |  |  |  |  |  |  |  |  |  |  |
| HCW30 | F | SD | < 65 | / | / | - |  |  |  |  |  |  |  |  |  |  |  |  |  |  |  |  |  |  |  |  |
| HCW31 | F | SD | < 65 | / | / | - |  |  |  |  |  |  |  |  |  |  |  |  |  |  |  |  |  |  |  |  |
| HCW32 | F | SD | < 65 | / | / | - |  |  |  |  |  |  |  |  |  |  |  |  |  |  |  |  |  |  |  |  |
| HCW33 | F | SD | < 65 | / | / | - |  |  |  |  |  |  |  |  |  |  |  |  |  |  |  |  |  |  |  |  |
| HCW34 | F | SD | < 65 | / | / | - |  |  |  |  |  |  |  |  |  |  |  |  |  |  |  |  |  |  |  |  |
| HCW35 | F | SD | < 65 | / | / | - |  |  |  |  |  |  |  |  |  |  |  |  |  |  |  |  |  |  |  |  |
| HCW36 | F | SD | < 65 | / | / | - |  |  |  |  |  |  |  |  |  |  |  |  |  |  |  |  |  |  |  |  |
| HCW37 | F | SD | < 65 | / | / | - |  |  |  |  |  |  |  |  |  |  |  |  |  |  |  |  |  |  |  |  |
| HCW38 | F | SD | < 65 | / | / | - |  |  |  |  |  |  |  |  |  |  |  |  |  |  |  |  |  |  |  |  |
| HCW39 | F | SD | < 65 | / | / | - |  |  |  |  |  |  |  |  |  |  |  |  |  |  |  |  |  |  |  |  |
| HCW40 | F | SD | < 65 | / | / | - |  |  |  |  |  |  |  |  |  |  |  |  |  |  |  |  |  |  |  |  |
| HCW41 | F | SD | < 65 | / | / | - |  |  |  |  |  |  |  |  |  |  |  |  |  |  |  |  |  |  |  |  |
| HCW42 | F | SD | < 65 | / | / | - |  |  |  |  |  |  |  |  |  |  |  |  |  |  |  |  |  |  |  |  |
| HCW43 | F | SD | < 65 | / | / | - |  |  |  |  |  |  |  |  |  |  |  |  |  |  |  |  |  |  |  |  |
| HCW44 | F | SD | < 65 | / | / | - |  |  |  |  |  |  |  |  |  |  |  |  |  |  |  |  |  |  |  |  |
| HCW45 | F | SD | < 65 | / | / | - |  |  |  |  |  |  |  |  |  |  |  |  |  |  |  |  |  |  |  |  |
| HCW46 | F | SD | < 65 | / | / | - |  |  |  |  |  |  |  |  |  |  |  |  |  |  |  |  |  |  |  |  |
| HCW47 | F | SD | < 65 | / | / | - |  |  |  |  |  |  |  |  |  |  |  |  |  |  |  |  |  |  |  |  |
| HCW48 | F | SD | < 65 | / | / | - |  |  |  |  |  |  |  |  |  |  |  |  |  |  |  |  |  |  |  |  |
| HCW49 | F | SD | < 65 | / | / | - |  |  |  |  |  |  |  |  |  |  |  |  |  |  |  |  |  |  |  |  |
| HCW50 | F | SD | < 65 | / | / | - |  |  |  |  |  |  |  |  |  |  |  |  |  |  |  |  |  |  |  |  |
| HCW51 | F | SD | < 65 | / | / | - |  |  |  |  |  |  |  |  |  |  |  |  |  |  |  |  |  |  |  |  |
| HCW52 | F | SD | < 65 | / | / | - |  |  |  |  |  |  |  |  |  |  |  |  |  |  |  |  |  |  |  |  |
| HCW53 | F | SD | < 65 | / | / | - |  |  |  |  |  |  |  |  |  |  |  |  |  |  |  |  |  |  |  |  |
| HCW54 | F | SD | < 65 | / | / | - |  |  |  |  |  |  |  |  |  |  |  |  |  |  |  |  |  |  |  |  |
| HCW55 | F | SD | < 65 | / | / | - |  |  |  |  |  |  |  |  |  |  |  |  |  |  |  |  |  |  |  |  |
| HCW56 | F | SD | < 65 | / | / | - |  |  |  |  |  |  |  |  |  |  |  |  |  |  |  |  |  |  |  |  |
| HCW57 | F | SD | < 65 | / | / | - |  |  |  |  |  |  |  |  |  |  |  |  |  |  |  |  |  |  |  |  |
| HCW58 | F | SD | < 65 | / | / | - |  |  |  |  |  |  |  |  |  |  |  |  |  |  |  |  |  |  |  |  |
| HCW59 | F | SD | < 65 | / | / | - |  |  |  |  |  |  |  |  |  |  |  |  |  |  |  |  |  |  |  |  |
| HCW60 | F | SD | < 65 | / | / | - |  |  |  |  |  |  |  |  |  |  |  |  |  |  |  |  |  |  |  |  |
| HCW61 | F | SD | < 65 | / | / | - |  |  |  |  |  |  |  |  |  |  |  |  |  |  |  |  |  |  |  |  |
| HCW62 | F | SD | < 65 | / | / | - |  |  |  |  |  |  |  |  |  |  |  |  |  |  |  |  |  |  |  |  |
| HCW63 | F | SD | < 65 | / | / | - |  |  |  |  |  |  |  |  |  |  |  |  |  |  |  |  |  |  |  |  |
| HCW64 | F | SD | < 65 | / | / | - |  |  |  |  |  |  |  |  |  |  |  |  |  |  |  |  |  |  |  |  |
| HCW65 | F | SD | < 65 | / | / | - |  |  |  |  |  |  |  |  |  |  |  |  |  |  |  |  |  |  |  |  |
| HCW66 | F | SD | < 65 | / | / | - |  |  |  |  |  |  |  |  |  |  |  |  |  |  |  |  |  |  |  |  |
| HCW67 | F | SD | < 65 | / | / | - |  |  |  |  |  |  |  |  |  |  |  |  |  |  |  |  |  |  |  |  |
| HCW68 | F | SD | < 65 | / | / | - |  |  |  |  |  |  |  |  |  |  |  |  |  |  |  |  |  |  |  |  |
| HCW69 | F | SD | < 65 | / | / | - |  |  |  |  |  |  |  |  |  |  |  |  |  |  |  |  |  |  |  |  |
| HCW70 | F | SD | < 65 | / | / | - |  |  |  |  |  |  |  |  |  |  |  |  |  |  |  |  |  |  |  |  |
| HCW71 | F | SD | < 65 | / | / | - |  |  |  |  |  |  |  |  |  |  |  |  |  |  |  |  |  |  |  |  |
| HCW72 | F | SD | < 65 | / | / | - |  |  |  |  |  |  |  |  |  |  |  |  |  |  |  |  |  |  |  |  |
| HCW73 | F | SD | < 65 | / | / | - |  |  |  |  |  |  |  |  |  |  |  |  |  |  |  |  |  |  |  |  |
| HCW74 | F | SD | < 65 | / | / | - |  |  |  |  |  |  |  |  |  |  |  |  |  |  |  |  |  |  |  |  |
| HCW75 | F | SD | < 65 | / | / | - |  |  |  |  |  |  |  |  |  |  |  |  |  |  |  |  |  |  |  |  |
| HCW76 | F | SD | < 65 | / | / | - |  |  |  |  |  |  |  |  |  |  |  |  |  |  |  |  |  |  |  |  |
| HCW77 | F | SD | < 65 | / | / | - |  |  |  |  |  |  |  |  |  |  |  |  |  |  |  |  |  |  |  |  |
| HCW78 | F | SD | < 65 | / | / | - |  |  |  |  |  |  |  |  |  |  |  |  |  |  |  |  |  |  |  |  |
| HCW79 | F | SD | < 65 | / | / | - |  |  |  |  |  |  |  |  |  |  |  |  |  |  |  |  |  |  |  |  |
| HCW80 | F | SD | < 65 | / | / | - |  |  |  |  |  |  |  |  |  |  |  |  |  |  |  |  |  |  |  |  |
| HCW81 | F | SD | < 65 | / | / | - |  |  |  |  |  |  |  |  |  |  |  |  |  |  |  |  |  |  |  |  |
| HCW82 | F | MD | < 65 | / | / | - |  |  |  |  |  |  |  |  |  |  |  |  |  |  |  |  |  |  |  |  |
| HCW83 | F | MD | < 65 | / | / | - |  |  |  |  |  |  |  |  |  |  |  |  |  |  |  |  |  |  |  |  |
| HCW84 | F | MD | < 65 | / | / | - |  |  |  |  |  |  |  |  |  |  |  |  |  |  |  |  |  |  |  |  |
| HCW85 | F | MD | < 65 | / | / | - |  |  |  |  |  |  |  |  |  |  |  |  |  |  |  |  |  |  |  |  |
| HCW86 | F | MD | < 65 | / | / | - |  |  |  |  |  |  |  |  |  |  |  |  |  |  |  |  |  |  |  |  |
| HCW87 | F | MD | < 65 | / | / | - |  |  |  |  |  |  |  |  |  |  |  |  |  |  |  |  |  |  |  |  |
| HCW88 | F | MD | < 65 | / | / | - |  |  |  |  |  |  |  |  |  |  |  |  |  |  |  |  |  |  |  |  |
| HCW89 | F | MD | < 65 | / | / | - |  |  |  |  |  |  |  |  |  |  |  |  |  |  |  |  |  |  |  |  |
| HCW90 | F | MD | < 65 | / | / | - |  |  |  |  |  |  |  |  |  |  |  |  |  |  |  |  |  |  |  |  |
| HCW91 | F | MD | < 65 | / | / | - |  |  |  |  |  |  |  |  |  |  |  |  |  |  |  |  |  |  |  |  |
| HCW92 | F | MD | < 65 | / | / | - |  |  |  |  |  |  |  |  |  |  |  |  |  |  |  |  |  |  |  |  |
| HCW93 | F | MD | < 65 | / | / | - |  |  |  |  |  |  |  |  |  |  |  |  |  |  |  |  |  |  |  |  |
| HCW94 | F | MD | < 65 | / | / | - |  |  |  |  |  |  |  |  |  |  |  |  |  |  |  |  |  |  |  |  |
| HCW95 | F | MD | < 65 | / | / | - |  |  |  |  |  |  |  |  |  |  |  |  |  |  |  |  |  |  |  |  |
| HCW96 | F | MD | < 65 | / | / | - |  |  |  |  |  |  |  |  |  |  |  |  |  |  |  |  |  |  |  |  |
| HCW97 | F | MD | < 65 | / | / | - |  |  |  |  |  |  |  |  |  |  |  |  |  |  |  |  |  |  |  |  |
| HCW98 | F | ED | < 65 | - | - | + | GEN, KAN, TOB, ERY, CLI, CIP | A | t001 | 5 | I | II | - |  |  |  |  |  |  |  |  |  |  |  |  |  |
| HCW99 | F | ED | < 65 | - | - | + | GEN, KAN, TOB, CHL | C | t242 | 5 | V | II | - |  |  |  |  |  |  |  |  |  |  |  |  |  |
| HCW100 | F | SD | < 65 | - | - | + | GEN, KAN, TOB, CIP | B | t041 | 5 | I | II | - |  |  |  |  |  |  |  |  |  |  |  |  |  |
| HCW101 | F | SD | < 65 | - | - | + | GEN, KAN, TOB, ERY, CLI, CIP, RIF, TET | D | t030 | 8 | III | I | - |  |  |  |  |  |  |  |  |  |  |  |  |  |
| HCW102 | F | SD | < 65 | - | - | + | susceptible to all tested antibiotics except beta-lactams | E | t595 | 152 | V | I | - |  |  |  |  |  |  |  |  |  |  |  |  |  |
| HCW103 | F | SD | < 65 | - | - | + | GEN, KAN, TOB, CHL | C | t242 | 5 | V | I | - |  |  |  |  |  |  |  |  |  |  |  |  |  |
| HCW104 | F | SD | < 65 | - | - | + | susceptible to all tested antibiotics except beta-lactams | F | t005 | 22 | V | I | - |  |  |  |  |  |  |  |  |  |  |  |  |  |
| HCW105 | F | SD | < 65 | - | - | + | KAN, FA | G | t044 | 80 | IV | III | + |  |  |  |  |  |  |  |  |  |  |  |  |  |

P, patient; HCW, healthcare worker; F, female; M, male, ED, emergency department; SD, surgical department; MD, medical department; S, surgical; NS, nonsurgical; CC, clonal complex; MLVA, Multiple-locus variable-number tandem-repeat assay; SCC*mec*, staphylococcal chromosome cassette *mec*; PVL, Panton–Valentine leukocidin; GEN, gentamicin; KAN, kanamycin; TOB, tobramycin; ERY, erythromycin; CLI, clindamycin; CIP, ciprofloxacin; RIF, rifampin; TET, tetracycline; CHL, chloramphenicol; FA, fusidic acid.
