# Supplementary material for: Carriage and Genetic Diversity of Methicillin-Resistant Staphylococcus aureus among Patients and Healthcare Workers in a Serbian University Hospital
Source: PLoS One. 2015 May 20;10(5):e0127347. doi: 10.1371/journal.pone.0127347 (PMC4439055; doi:10.1371/journal.pone.0127347)
Supplement: S2 Table — Presence of methicillin-resistant Staphylococcus aureus (MRSA) strains among study population (patients and healthcare workers) in nasal and/or throat samples and growth of MRSA strains on culture media. (DOCX) [file pone.0127347.s002.docx]

Table S2. Raw data. Presence of methicillin-resistant *Staphylococcus aureus* (MRSA) strains among study population (patients and healthcare workers) in nasal and/or throat samples and growth of MRSA strains on culture media.

| Origin of MRSA strain (patient / HCW) | Origin of MRSA strain  (nose/throat) | Growth on MRSA-ID after 24h | Growth on MRSA-ID after 48h | Growth on ORSA after 24h | Growth on ORSA after 24h | Growth on MSA-oxacillin after 24h | Growth on MSA-oxacillin after 48h |
| --- | --- | --- | --- | --- | --- | --- | --- |
| P 173 | nose | + | + | + | + | + | + |
|  | throat | + | + | + | + | + | + |
| P 174 | nose | + | + | + | + | + | + |
|  | throat | + | + | + | + | + | + |
| P 175 | throat | + | + | + | + | + | + |
| P 176 | nose | + | + | - | - | - | + |
|  | throat | + | + | + | + | + | + |
| P 177 | throat | + | + | + | + | + | + |
| P 178 | throat | + | + | + | + | + | + |
| P 179 | throat | + | + | - | + | - | + |
| P 180 | throat | + | + | + | + | + | + |
| P 181 | nose | + | + | + | + | + | + |
|  | throat | - | + | + | + | + | + |
| P 182 | throat | + | + | + | + | + | + |
| P 183 | nose | + | + | + | + | + | + |
|  | throat | + | + | + | + | + | + |
| P 184 | nose | + | + | + | + | + | + |
|  | throat | + | + | + | + | + | + |
| P 185 | nose | - | + | - | + | - | - |
|  | throat | + | + | + | + | + | + |
| P 186 | nose | - | + | - | + | - | - |
| P 187 | throat | + | + | + | + | + | + |
| P 188 | nose | + | + | - | - | - | - |
| P 189 | nose | - | - | + | + | - | + |
| P 190 | nose | + | + | - | - | + | + |
| P 191 | nose | + | + | - | + | - | + |
| P 192 | nose | - | + | + | + | + | + |
| P 193 | throat | + | + | - | - | - | - |
| P 194 | nose | - | - | + | + | + | + |
|  | throat | + | + | + | + | + | + |
| P 195 | nose | + | + | + | + | + | + |
|  | throat | + | + | + | + | + | + |
| HCW 98 | nose | - | + | + | + | + | + |
| HCW 99 | nose | + | + | - | - | - | - |
| HCW 100 | throat | + | + | + | + | + | + |
| HCW 101 | throat | + | + | + | + | + | + |
| HCW 102 | nose | + | + | - | + | - | - |
|  | throat | + | + | - | - | - | - |
| HCW 103 | nose | + | + | - | - | - | - |
| HCW 104 | throat | - | + | + | + | - | + |
| HCW 105 | nose | + | + | + | + | - | + |

P, patient; HCW, healthcare workers; ORSA, oxacillin resistance screening agar; MSA-oxacillin, mannitol salt agar supplemented with 2 mg/L of oxacillin.
